# Health e-Cards as a Means of Encouraging Help Seeking for Depression Among Young Adults: Randomized Controlled Trial

**DOI:** 10.2196/jmir.1294

**Published:** 2009-10-22

**Authors:** Daniel L Costin, Andrew J Mackinnon, Kathleen M Griffiths, Philip J Batterham, Anthony J Bennett, Kylie Bennett, Helen Christensen

**Affiliations:** ^1^Centre for Mental Health ResearchThe Australian National UniversityCanberra, ACTAustralia; ^2^Department of PsychologyThe Australian National UniversityCanberra, ACTAustralia; ^3^Orygen Research CentreUniversity of MelbourneParkville, VicAustralia

**Keywords:** Depression, mood disorders, young adult, health care seeking behavior, attitude to health, intention, health promotion, randomized controlled trial, electronic mail

## Abstract

**Background:**

There is a need to identify interventions that increase help seeking for depression among young adults.

**Objective:**

The aim was to evaluate a brief depression information intervention employing health e-cards (personalized emails containing links to health information presented on a Web page).

**Methods:**

A randomized controlled trial was carried out with 348 19- to 24-year-olds drawn from the community. Participants were randomized to receive one of three conditions, all of which delivered a short series of health e-cards. Two active conditions involved the delivery of depression information designed to increase help-seeking behavior and intentions and to improve beliefs and knowledge associated with help seeking. A control arm delivered information about general health issues unrelated to depression. The primary outcome was help-seeking behavior. Secondary outcomes were help-seeking intentions; beliefs about the efficacy of depression treatments and help sources; ability to recognize depression; knowledge of the help-seeking process; and depressive symptoms. The study’s primary focus was outcomes relating to formal help seeking (consultation with a general practitioner or mental health professional) but also targeted behaviors, intentions, and beliefs relating to informal help seeking.

**Results:**

Relative to the control condition, depression health e-cards were not associated with an increase in formal help-seeking behavior, nor were they associated with improved beliefs about depression treatments; ability to recognize depression; knowledge of the help-seeking process; or depressive symptoms. Depression e-cards were associated with improved beliefs about the overall efficacy of formal help sources (*z* = 2.4, *P* = .02). At post-intervention, participants in all conditions, relative to pre-intervention, were more likely to have higher intentions of seeking help for depression from a formal help source (*t*_641_ = 5.8, *P* < .001) and were more likely to rate interpersonal psychotherapy as being helpful (*z* = 2.0, *P* = .047). Depression e-cards were not associated with any significant changes in informal help-seeking behavior, intentions, or beliefs.

**Conclusions:**

The study found no evidence that providing depression information in the form of brief e-cards encourages help seeking for depression among young adults. Involvement in the study may have been associated with increased help-seeking intentions among participants in all conditions, suggesting that mechanisms other than depression information may increase help seeking.

**Trial Registration:**

International Standard Randomized Controlled Trial Number (ISRCTN): ISRCTN98406912; http://www.controlled-trials.com/ISRCTN98406912/ISRCTN98406912 (Archived by WebCite at http://www.webcitation.org/5k221KiMi)

## Introduction

Depression is a leading contributor to the burden of disease and injury among young adults [1]. Around 6.4% of young Australians aged 18-24 years will be affected by depression each year [1]. Many young adults are unable to recognize the symptoms of depression [2], have limited knowledge about appropriate treatment options [3], and are reluctant to seek help for depression from a general practitioner (GP) or mental health professional [4]. As a consequence, their depression may go unrecognized and untreated. There is a need to develop and evaluate interventions that educate young adults about depression and encourage those experiencing symptoms to consult a health professional.

To date, only three studies have used a randomized controlled trial to investigate depression informational interventions for their effects on help-seeking behavior. Jorm et al [5] provided depressed people in the community with an evidence-based consumer guide to treatments for depression and found that participants who received this guide were no more likely to report subsequently seeking professional help for depression than control group participants who received a short brochure about depression (a rise in help seeking was observed in both groups). A major weakness of this study was the lack of an appropriate comparator—the brochure used in the control condition also provided information about evidence-based treatments for depression. Christensen et al [6] examined the effect of a 6-week intervention involving access to a depression information website and weekly telephone contact from a lay interviewer who directed participants to read particular sections of the website. Relative to an attention control condition (brief weekly telephone contact with a lay interviewer who asked questions about factors that might affect depression, but no access to the website), participants in the website condition were no more likely to report an increase in help seeking from GPs or mental health professionals at 6 months follow-up. A limitation of this study was that participants were recruited to an early intervention trial, so their agreement to participate in the trial might in itself be regarded as an act of help seeking. Finally, in the only randomized controlled trial to have investigated depression information interventions for their effects on help-seeking behavior in young adults, Sharpe et al [7] examined the effect of a 40-minute, classroom-based intervention delivered to university students. They found that the intervention led to more positive attitudes toward seeking psychological help but had no effect on help-seeking behavior compared to an attention placebo condition. A limitation of this study was that participants were not selected on the basis of having elevated levels of psychological distress or depression, so it is difficult to determine whether the failure to modify help-seeking behavior was due to the intervention or low need for help seeking in this sample.

The current study sought to address some of the limitations of these previous studies [5-7] and to add to the evidence about the effectiveness of brief information interventions in increasing help seeking for depression among young adults. Given the convenience and low cost of disseminating public health interventions on the Internet [8] and the popularity of this medium for young people [9], the study employed an Internet intervention. In addition, since it might be anticipated that brief rather than more-extensive Internet interventions would be preferred by young people [10], the intervention employed “health e-cards,” an electronic analogue to postcards. A health e-card is a personal email containing a link to depression information presented on a Web page. Previous research has demonstrated that postcards can be an effective means of reducing incidences of self-harm [11], and thus the brief e-cards were considered an appropriate modality in the current context.

Participants were randomized to receive one of three conditions, all of which delivered a short series of health e-cards. Two active conditions (a basic and an enhanced condition) involved the delivery of depression information, and one control arm (control) delivered information about general health issues.

The active conditions were designed to facilitate progression of the process that young people are likely to go through when seeking help for mental health problems. This help seeking process, as conceptualized by Rickwood and colleagues [12], begins with the awareness of symptoms and appraisal of having a problem that may require intervention. The young person must then be able to articulate his or her problem using words that he or she feels comfortable using and that will be understood by others. Finally, sources of help must be available and accessible, and the help seeker must be willing to seek out and disclose information to these sources [12]. The information provided in the active conditions aimed to modify factors that might inhibit progression through this help-seeking process, specifically, the inability to recognize depression [13], negative attitudes toward health professionals and the treatments they provide [14], and lack of knowledge and understanding of where to seek professional help, the services available, how to contact them [15,16], and what to expect at a consultation [12].

To address limitations of previous studies, the control arm provided information about general health issues that were likely to be of relevance to young adults but not related to depression [5]. Furthermore, the trial was presented to participants as a health and well-being trial rather than one that explicitly concerned depression, and the screening and pre-intervention survey included placebo questions relating to general health conditions [6]. Finally, the intervention was delivered to young adults drawn from the community, and the sample was stratified to include individuals experiencing high levels of distress who could be considered in need of help [7].

We hypothesized that, relative to the control group, individuals in the two active conditions would be more likely to seek help for depression from a GP or mental health professional over 3 subsequent weeks. We also hypothesized that participants in the active arms would report a greater willingness to seek professional help for depression in the future, report more positive beliefs about the efficacy of treatments and health professionals, exhibit improved ability to recognize depressive symptoms, demonstrate improvements in their knowledge of the help-seeking process, and have reduced depressive symptoms. The study’s primary focus was outcomes relating to formal help seeking (consultation with a GP or mental health professional), but it also targeted behaviors, intentions, and beliefs relating to informal help seeking (seeking assistance from peers and family).

## Methods

### Participants and Flow

**Figure 1:**
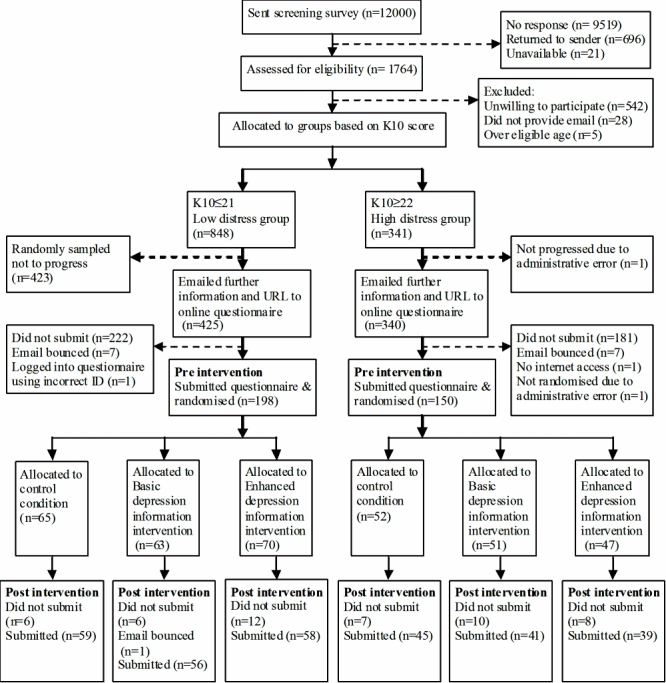
Flow of participants

Figure 1 details the flow of participants through the trial. Participants were recruited by means of a screening questionnaire, posted in March 2007 to 12,000 individuals aged 19 to 23 selected at random from the Australian Electoral Roll. Registration on the electoral roll is compulsory in Australia. The response rate was 14.7% (1764/12000). Respondents were eligible for inclusion in the trial if they indicated a willingness to receive further information about participating in the trial, provided their first name and email address, and fell within the age range of 19-24 years.

Two strata were formed with the intention to conduct a priori subgroup analyses. The first group consisted of individuals experiencing high levels of distress who could be considered in need of help. The second consisted of individuals experiencing lower levels of distress or none at all. Although the second group may not have personally required help, the prevalence of depression is such that there was a high likelihood that individuals in this group might need to recognize and encourage help seeking in a friend (around 18% of young adults report that they would seek help for depression from a friend rather than a health professional [4]).

Eligible respondents scoring 22 or higher on the Kessler Psychological Distress Scale (K10) [17] formed the high distress group, and those scoring 21 or lower formed the low distress group. We anticipated that at least two thirds of eligible respondents would fall into the low distress group. To yield more equal numbers of high and low distress participants in the intervention, we randomly sampled members of the low distress group so that only 50% progressed to the next stage of recruitment, but all members of the high distress group progressed to the next stage. Individuals who progressed were sent further information by email and an URL link to the online pre-intervention questionnaire. The study was approved by the Australian National University Human Research Ethics Committee.

### Treatment Allocation

Participants were randomized to conditions after submitting the online pre-intervention questionnaire using block randomization with computer-generated random numbers. Randomization was stratified by sex and pre-intervention score on the Center for Epidemiological Studies Depression Scale (CES-D) [18]. The randomization tables were set up prior to commencement of the study. Randomization was carried out by one of the authors (DC).

### Intervention

**Figure 2:**
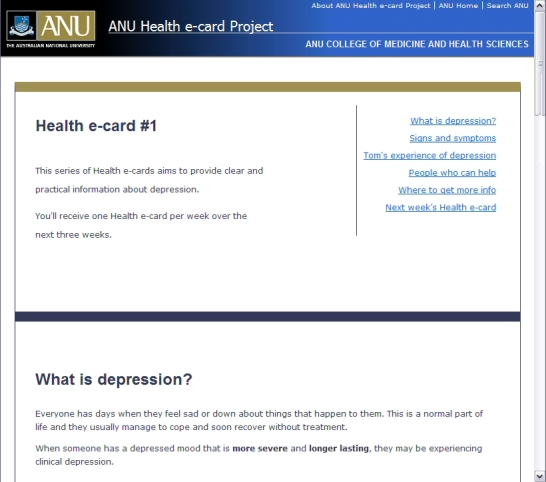
Screenshot of a health e-card

Individuals started the intervention within 7 days of submitting their pre-intervention questionnaire. The intervention consisted of three personalized emails containing an embedded URL to brief information presented on a Web page. These were described as health e-cards (see Figure 2). Each week for 3 weeks, participants received an automated email from the project coordinator. Emails were personalized with the participant’s first name and contained a greeting and directions to click on an URL link to view the brief health information (see Multimedia Appendix). The health e-cards were designed to be read as a series, much like an e-learning program.

Participants in the basic intervention received depression information health e-cards. The information provided included symptoms of depression, a vignette describing a young man’s experience of depression, where to find evidence-based information about depression and its treatment on the Internet, prevalence rates among 18- to 24-year-olds, encouragement to consult with a health professional if feeling depressed, and information about GPs, counselors, clinical psychologists, and psychiatrists, including who they are, treatments they provide, and how to locate them.

Participants in the enhanced intervention also received depression information health e-cards. In addition to the information provided in the basic intervention, these participants also received information on facts about depression and help seeking, what to expect at an initial consultation with each health professional, and practical tips about making contact with health professionals and asking for help. It was anticipated that this additional information would help facilitate movement through the help-seeking process [12] and enhance the effect of depression information in modifying help-seeking behavior [19].

Participants in the control condition received health e-cards containing information on health issues not directly related to depression but still relevant to young adults. These were meningococcal disease, amphetamines, and gamma-hydroxybutyrate (GHB).

The treatments for depression recommended in the basic and enhanced interventions were antidepressants, cognitive behavioral therapy, and interpersonal psychotherapy. These recommendations were based on systematic reviews [20-23]. Information relating to GPs was sourced from Ellis and Smith [24], and information relating to the role and qualifications of different health professionals was sourced from The Australian Psychological Society’s website [25]. Information provided in the control condition was reproduced with permission from other health promotion publications [26-29].

### Post-Intervention Data Collection

At 3 weeks post-intervention, the automatic email application sent participants a personalized email with an URL link to the post-intervention questionnaire. This email was resent up to three times at 1-week intervals, or until the participant’s post-intervention questionnaire was submitted. To our knowledge, there are no data available on the average period of time it takes to complete a visit with a health professional after forming the intention to seek help for depression. We estimated that a period of 3 weeks would be sufficient and would also maximize post-intervention response rates.

### Measures

#### Demographic Information

At screening, data were collected on sex, age, employment status, education, email usage, demographic characteristics based on residential post code, experience seeking help for depression from a GP or mental health professional (General Help Seeking Questionnaire [GHSQ] supplementary questions) [30], current help seeking, and psychological distress (as measured by the K10 [17]).

#### Primary Outcome Measure: Help-Seeking Behavior

The primary outcome was the proportion of participants who reported at post-intervention that they had sought help for feelings of depression from a formal source (GP or mental health professional) in the past 6 weeks, assessed with the Actual Help Seeking Questionnaire (AHSQ) [12]. Participants were also asked whether they had sought help from an informal help source (friend, partner, or family member).

#### Secondary Outcome Measures

Secondary outcomes were collected at baseline (screening or pre-intervention) and post-intervention.

Intentions to seek help were assessed using the GHSQ [30]. Respondents were asked, “If you were feeling depressed, how likely is it that you would seek help or advice from the following people during the next six weeks?” They rated, on a 7-point scale (1 = extremely unlikely to 7 = extremely likely), their intentions to seek help from a friend, partner, family member, GP, counselor, clinical psychologist, psychiatrist, or no one. An optional item, “someone else not listed above (please describe who this is),” was also included. Two multi-item scales were created for the analysis using methods described by Deane and Wilson [31]. These were intentions to seek formal help (mean of responses for GP, counselor, clinical psychologist, and psychiatrist) and intentions to seek informal help (mean of responses for friend, partner, and family member). The range of these multi-item scales was therefore 1 to 7, with higher scores representing stronger intentions to seek help.

Beliefs about help seeking were assessed using a modified version of a measure used by Jorm et al [32]. Respondents were asked to rate the helpfulness of various formal help sources (GPs, counselors, clinical psychologists, and psychiatrists), informal help sources (partner, family, and friends), and treatments (antidepressants, cognitive behavioral therapy, interpersonal psychotherapy, and supportive counseling) for a young adult experiencing depression. Responses included “helpful,” “harmful,” “neither helpful nor harmful,” or “don’t know.” Results were analyzed as the proportion of respondents rating each help source or treatment as “helpful.”

Ability to recognize depression was assessed by presenting a vignette describing a 23-year-old male with major depression [32] according to the *Diagnostic and Statistical Manual of Mental Disorders, Fourth Edition* (DSM-IV). The character’s name and age was changed from Jorm and colleagues’ original version to make it more applicable to the participants in this study. Respondents were asked to indicate what (if anything) was wrong with the character by selecting one of 14 responses. The response list included the top 12 responses given by Australian participants in a previous study [33] plus two additional items (bulimia nervosa or meningococcal disease) relating to placebo questions included in the screening survey. A correct response was considered to be one in which the participant endorsed depression.

Help-seeking knowledge was assessed by asking participants to do the following: “Imagine a good mate or close friend came to you and said that they had been feeling depressed for several weeks and were thinking about going to see a health professional but didn’t know much about it, or where to get help.” They then rated how much they agreed with each of three statements using a 5-point scale (4 = strongly agree, 3 = agree, 2 = neither agree nor disagree, 1 = disagree, 0 = strongly disagree). The statements were as follows: “I would be able to explain to my friend the type of help or treatment that the following health professionals generally provide for people who have depression,” “I would be able to explain to my friend how to locate and contact these health professionals,” and “I would be able to explain to my friend what to expect at an initial consultation with these health professionals.” The health professionals listed were GPs, counselors, clinical psychologists, and psychiatrists. Ratings for each health professional were cumulated across the three statements to produce four subscores. These were perceived knowledge about seeking help from GPs, counselors, clinical psychologists, and psychiatrists. The range for each subscore was 0-12. A total score was also produced (sum of all items), which ranged from 0-48.

Symptoms of depression were assessed using the CES-D [18]. Higher scores represent greater psychological distress, with scores of 16 or higher usually taken to indicate clinical depression.

To assess the appeal of depression health e-cards, screening survey respondents were asked what type of health information they thought would be most helpful for inclusion in health e-cards aimed at young adults. There were 26 different topics offered, and respondents selected as many items as they wished. Furthermore, at post-intervention, participants were asked to rate the helpfulness of each e-card (very helpful, helpful, neither helpful nor unhelpful, unhelpful, or did not read).

### Statistical Methods

Demographic characteristics were compared using one-way analysis of variance (ANOVA) and tests of association (chi-square). Three sets of analyses were conducted: (1) an analysis of differences between the three conditions within the low distress group, (2) an analysis of differences between the three conditions within the high distress group, and (3) an analysis of differences between the high distress group and the low distress group.

Formal help-seeking behavior and informal help-seeking behavior were examined using logistic regression. Predictor variables were condition, distress group, and interaction of condition and distress group. Participants who did not respond to the post-intervention questionnaire were presumed not to have sought help. Ordinal repeated measure outcomes were analyzed using linear mixed models in SPSS 15.0 (SPSS Inc, Chicago, IL, USA ), and nominal repeated measure outcomes were analyzed using mixed logit models in Stata 10 (StataCorp LP, College Station, TX, USA). The analyses examined for main effects of condition, wave (pre-intervention vs post-intervention), and distress group; two-way interaction effect of condition and wave; and three-way interaction effect of condition, wave, and distress group. The major significant effect sought was an interaction of condition and wave that would indicate the effect of condition over time in increasing help-seeking intentions, beliefs about help seeking, ability to recognize depression, and help-seeking knowledge and in decreasing symptoms of depression. The interaction between distress group and condition was also of potential interest (whether the intervention was more useful for those with high levels of distress compared to those with low levels of distress). However, the three-way interaction between condition, wave, and distress group was not significantly associated with any of the outcomes, nor was the interaction between distress group and condition. Consequently, the final models included only the main effects and the interaction effect of condition and wave. All effects were tested at the *P* < .05 level.

### Power Analysis

Target sample size was determined using GP attendance data previously analyzed by Parslow et al [34] from which a baseline consultation rate of 15% over the 6 weeks of the study was estimated. Consultation rates of between 10% and 20% arising from the invention were deemed possible. To maintain power at 80%, we sought to recruit 80 high distress participants into each arm of the trial. This sample would have comparable power to detect moderate-sized differences (less than .5 standard deviations) between control and active arms for secondary outcomes measured on continuous scales. Accordingly, the same target was set for the low distress group.

## Results

The post-intervention response rate was 85.6% (298/348). Demographic characteristics are presented in Table 1. A preliminary analysis of the effect of condition on outcome variables revealed no significant differences between the two active arms (basic and enhanced), so these were combined into a single “depression information” condition for subsequent analyses.

**Table 1:** Demographic characteristics^a,b^

		Low Distress Group			High Distress Group		
		Control (n = 65)	Basic (n = 63)	Enhanced (n = 70)	Control (n = 52)	Basic (n = 51)	Enhanced (n = 47)
Age, years, mean (SD)		21.6 (1.6)	21.6 (1.4)	21.3 (1.4)	21.4 (1.5)	21.1 (1.3)	21.4 (1.6)
**Sex**							
	Male	19 (29.2)	16 (25.4)	21 (30.0)	8 (15.4)	11 (21.6)	3 (6.4)
	Female^c^	46 (70.8)	47 (74.6)	49 (70.0)	44 (87.6)	40 (78.4)	44 (93.6)
**Highest level of education completed**							
	Less than year 12	1 (1.54)	4 (6.35)	4 (5.71)	5 (9.62)	4 (7.84)	3 (6.38)
	Year 12^c^	22 (33.8)	24 (38.1)	23 (32.9)	20 (38.5)	30 (58.8)	25 (53.2)
	Year 12+ (certificate I-IV or diploma)	21 (32.3)	15 (23.8)	21 (30.0)	14 (26.9)	12 (23.5)	10 (21.3)
	Bachelor degree or higher^c^	21 (32.3)	20 (31.7)	22 (31.4)	13 (25.0)	5 (9.8)	9 (19.1)
**Highest current studies**							
	Not studying^c^	26 (40.0)	20 (31.7)	30 (42.9)	9 (17.3)	12 (23.5)	12 (25.5)
	Higher secondary certificate	0 (0.0)	0 (0.0)	0 (0.0)	1 (1.9)	0 (0.0)	1 (2.1)
	Certificate I-IV or diploma^c^	7 (10.8)	6 (9.5)	5 (7.1)	9 (17.3)	9 (17.6)	8 (17.0)
	Bachelor degree or higher	32 (49.2)	37 (58.7)	34 (48.6)	33 (63.5)	29 (56.9)	26 (55.3)
**Employment status**							
	Employed full-time	28 (43.1)	24 (38.1)	31 (44.3)	21 (40.4)	14 (27.5)	14 (29.8)
	Employed part-time/casual	25 (38.5)	27 (42.9)	29 (41.4)	26 (50.0)	29 (56.9)	23 (48.9)
	Not currently employed	12 (18.5)	12 (19.0)	10 (14.3)	5 (9.6)	8 (15.7)	10 (21.3)
**Demographic rating**							
	Metropolitan	38 (58.5)	35 (56.5)	38 (55.1)	29 (55.8)	26 (51.0)	20 (42.6)
	Provincial	17 (26.2)	17 (27.4)	19 (27.5)	19 (36.5)	16 (31.4)	14 (29.8)
	Rural	10 (15.4)	10 (16.1)	12 (17.4)	4 (7.7)	9 (17.6)	13 (27.7)
**Previous help-seeking for depression**							
	Previously^c^ sought help	18 (27.7)	14 (22.2)	15 (21.4)	26 (50.0)	29 (56.9)	28 (59.6)
	Rated helpfulness of the visits, mean (SD)	2 (0.7)	1.4 (0.9)	2.3 (1.4)	2 (0.9)	2.6 (1.5)	1.9 (0.8)
Currently receiving care for depression from GP or mental health professional^c^		4 (6.2)	1 (1.6)	3 (4.3)	11 (21.2)	12 (23.5)	10 (21.3)
Score on K10, mean (SD)^c^		16.1 (2.7)	15.5 (3.3)	15.6 (2.8)	27.0 (5.0)	26.9 (4.5)	27.3 (4.8)
Score on CES-D, mean (SD)^c^		10.2 (6.2)	10.2 (6.8)	10.0 (9.4)	21.8 (9.3)	22.4(10.0)	22.6 (9.8)
**Post-intervention response rates**							
	Did not submit questionnaire	6 (9.2)	6 (9.7)	12 (17.1)	7 (13.5)	10 (19.6)	8 (17.0)
	Completed questionnaire	59 (90.8)	56 (90.3)	58 (82.9)	45 (86.5)	41 (80.4)	39 (83.0)

^a^ All values are number (%) unless otherwise indicated.

^b^ Not all participants completed all questions.

^c^ Significant difference between distress groups (but not between conditions).

### Intervention Adherence

The embedded URL links that were emailed to participants ended with a unique identifier. This allowed us to track an individual’s adherence to delivered materials by analysing the Web logs for each health e-card site. Of 348 participants, 320 (92%) visited at least one health e-card site, 167 (48%) visited all three sites, 102 (29.3%) visited two sites, 51 (14.7%) visited one site, and 28 (8%) visited no sites. Intervention condition had no significant effect on the average number of sites visited by participants in the low distress group (F_1,196_ = .037, *P* = .85) or the high distress group (F_1,148_ = .097, *P* = .76).

### Primary Outcome: Help-Seeking Behavior

Table 2 presents the percentage of participants in each condition who reported seeking help for depression at post-intervention. Based on a logistic regression model, the interaction between condition and distress group was not significantly associated with help seeking from formal sources (OR = 0.69, *χ* ^2^_1_ = 0.15, *P* = .69) or from informal sources (OR = 2.25, *χ* ^2^_1_ = 2.83, *P* = .09). Participants in the high distress group were more likely than participants in the low distress group to report help seeking from formal (OR = 6.67, *χ* ^2^_1_ = 12.97, *P* < .001) and informal sources (OR = 5.55, *χ* ^2^_1_ = 34.10, *P* < .001). There was no effect of the intervention on help seeking from formal (OR = 1.17, *χ* ^2^_1_ = 0.14, *P* = .70) or informal (OR = 0.86, *χ* ^2^_1_ = 0.18, *P* = .67) sources.

**Table 2:** Primary outcome: percentage of participants in each condition seeking help from formal and informal sources up to 6 weeks after the intervention

	No.	Help-Seeking Source	
		Formal	Informal
**High distress group**			
Intervention	80	25.0%	75.0%
Control	45	26.7%	66.7%
Difference (95% CI)		−1.7% (−18.2% to 13.3%)	8.3% (−7.6% to 25.1%)
**Low distress group**			
Intervention	114	4.4%	25.4%
Control	59	3.4%	39.0%
Difference (95% CI)		−1.0% (−7.0% to 7.5%)	13.5% (−0.8% to 28.1%)

### Secondary Outcomes

Changes in secondary outcomes are presented in Table 3 and discussed below.

#### Help-Seeking Intentions

The interaction between condition and wave was not significantly associated with help-seeking intentions. At post-intervention, participants in both conditions had higher intentions of seeking help for depression from a formal source (*t*_641_ = 5.8, *P* < .001). At pre- and post-intervention, the high distress group, relative to the low distress group, had higher informal help-seeking intentions (*t*_641_ = 5.4, *P* < .001).

#### Beliefs About the Efficacy of Formal Help Sources

At post-intervention, individuals in the depression information condition, relative to the control condition, were more likely to rate at least one health professional as helpful (*z* = 2.4, *P* = .02). At pre- and post-intervention, individuals in the high distress group, relative to the low distress group, were less likely to rate GPs (*z* = −2.5, *P* = .01) and counselors (*z* = −3.1, *P* = .002) as helpful.

#### Beliefs About the Efficacy of Informal Help Sources

The interaction between condition and wave was not significantly associated with beliefs about the efficacy of informal help sources. At pre- and post-intervention, individuals in the high distress group, relative to the low distress group, were less likely to rate friend (*z* = −2.7, *P* = .007), partner (*z* = −3.3, *P* = .001), family member (*z* = −2.8, *P* = .005), or any informal source (*z* = −2.3, *P* = .02) as being helpful.

#### Beliefs About the Efficacy of Treatments

The interaction between condition and wave was not significantly associated with beliefs about the efficacy of treatments. At post-intervention, participants in both conditions were more likely to rate interpersonal psychotherapy as helpful (*z* = 2.0, *P* = .047).

#### Ability to Recognize Depression

The proportion of respondents correctly identifying the vignette as “depression” did not differ across conditions, distress groups, or waves.  There was no interaction between condition and wave (*z* = 0.1, *P* = .92).

#### Help-Seeking Knowledge

The interaction between condition and wave was not significantly associated with help-seeking knowledge. At pre- and post-intervention, individuals in the depression information condition, relative to the control condition, indicated that they knew more about seeking help from GPs (*t*_641_ = −2.3, *P* = .02). Individuals in the high distress group, relative to the low distress group, indicated that they knew more about seeking help from GPs (*t*_641_ = −2.2, *P* = .03), clinical psychologists (*t*_641_ = −2.0, *P* = .046), and all health professionals (total score; *t*_641_ = −2.5, *P* = .01). Significant effects of wave were found for GPs (*t*_641_ = −3.1, *P* = .002), clinical psychologists (*t*_641_ = −2.8, *P* = .006), psychiatrists (*t*_641_ = −3.0, *P* = .003), and all professionals (*t*_641_ = −3.6, *P* < .001), indicating that participants in both conditions were more knowledgeable at post-intervention about seeking help from these sources.

#### Symptoms of Depression

Symptoms of depression did not differ between pre- and post-intervention (*t*_641_ = 1.6, *P* = .11). There was no interaction between condition and wave. As expected, individuals in the high distress group, relative to the low distress group, scored higher on the CES-D at both pre- and post-intervention (*t*_641_ = −18.6, *P* < .001). A subanalysis was conducted to determine whether the intervention reduced symptoms of depression among the high distress group. No significant effects or interactions were found.

### Appeal of Depression Health e-Cards

Most (1598/1764, 90.6%) of the screening survey respondents selected “depression” when asked what type of health information they thought would be most helpful for inclusion in health e-cards aimed at young adults. Depression was the most frequently endorsed item.

At post-intervention, most participants rated the e-cards as “helpful” or “very helpful:” depression information condition: health e-card 1 (87.6%), health e-card 2 (83.0%), health e-card 3 (85.6%); control condition: health e-card 1 (90.4%), health e-card 2 (92.2%), health e-card 3 (91.0%).

### Subsidiary Analysis

Of participants in the high distress group, 22% were already receiving care for depression at baseline, and a high proportion of participants in both groups had previously sought help for depression from a health professional (24% of participants in the low distress group and 53% in the high distress group). To address this, the analysis of help-seeking behavior was repeated twice, first with the exclusion of participants who were receiving treatment for depression at baseline (n = 263), and then with the exclusion of this group as well as participants who had previously sought help for depression (n = 189). The results from both analyses were consistent with the primary analysis in that there was no significant effect of the intervention and the interaction between intervention condition and distress group was not significantly associated with formal or informal help seeking in either subgroup.

**Table 3:** Secondary outcomes^a^

		Low Distress Group				High Distress Group			
		Control		Intervention		Control		Intervention	
		Pre (n = 65)	Post (n = 59)	Pre (n = 133)	Post (n = 114)	Pre (n = 52)	Post (n = 45)	Pre (n = 98)	Post (n = 80)
**Help-seeking intentions**									
	Formal help source, mean (SD)^b^	2.82 (1.57)	3.16 (1.42)	2.47 (1.51)	3.40 (1.58)	2.71 (1.53)	3.36 (1.47)	2.43 (1.51)	3.21 (1.68)
	Informal help source, mean (SD)^c^	4.86 (1.41)	4.86 (1.60)	4.83 (1.64)	4.79 (1.64)	3.58 (1.52)	4.54 (1.74)	3.98 (1.72)	4.42 (1.55)
**Beliefs about formal help sources**									
	Rated GPs as helpful^c^	43 (66.20)	43 (72.90)	88 (66.20)	90 (78.90)	30 (57.70)	27 (60.00)	50 (51.00)	57 (71.30)
	Rated counselors as helpful^c^	54 (83.10)	51 (86.40)	102 (76.70)	101 (88.60)	37 (71.20)	34 (75.60)	61 (62.20)	62 (77.50)
	Rated clinical psychologists as helpful	41 (63.10)	41 (95.50)	98 (73.70)	83 (72.80)	36 (69.20)	28 (62.20)	66 (67.30)	62 (77.50)
	Rated psychiatrists as helpful	34 (52.30)	35 (59.30)	83 (62.40)	80 (70.20)	28 (53.80)	26 (57.80)	63 (64.30)	56 (70.00)
	Rated any formal source as helpful^d^	59 (90.80)	55 (93.20)	119 (89.50)	108 (94.70)	45 (86.50)	36 (80.00)	81 (82.70)	76 (95.00)
**Beliefs about informal help sources**									
	Rated friends as helpful^c^	59 (90.80)	56 (94.90)	110 (82.70)	90 (78.90)	40 (76.90)	32 (71.10)	71 (72.40)	64 (80.00)
	Rated partner rated as helpful^c^	59 (90.80)	93 (55.00)	119 (90.20)	95 (83.30)	34 (66.70)	34 (75.60)	73 (74.50)	70 (87.50)
	Rated family as helpful^c^	58 (89.20)	48 (81.40)	109 (82.60)	92 (80.70)	34 (66.70)	30 (66.70)	72 (73.50)	61 (76.30)
	Rated any informal source as helpful^c^	62 (95.40)	58 (98.30)	130 (97.70)	105 (82.10)	48 (92.30)	40 (88.90)	87 (88.80)	74 (92.50)
**Beliefs about treatments**									
	Rated antidepressants as helpful	34 (52.30)	37 (62.70)	69 (51.90)	60 (52.60)	25 (48.10)	23 (51.10)	53 (54.10)	50 (62.50)
	Rated CBT as helpful	36 (55.40)	34 (57.60)	60 (45.10)	64 (56.10)	24 (46.20)	21 (46.70)	44 (44.90)	47 (58.80)
	Rated IPT as helpful^b^	27 (41.50)	31 (52.50)	54 (40.60)	63 (55.30)	19 (36.50)	21 (46.70)	40 (40.80)	39 (48.80)
	Rated supportive counselling as helpful	56 (86.20)	51 (86.40)	113 (85.00)	101 (88.60)	45 (86.50)	35 (77.80)	86 (87.80)	67 (83.80)
**Ability to recognize depression**									
	Endorsed problem in vignette as depression	63 (96.90)	53 (89.80)	121 (91.00)	101 (88.60)	47 (90.40)	44 (97.80)	91 (92.90)	76 (72.50)
**Perceived knowledge of help-seeking process**									
	All health professionals, mean (SD)^b,c^	27.52 (11.17)	31.07 (10.35)	27.98 (10.71)	32.28 (9.38)	29.21 (11.29)	30.36 (11.21)	31.54 (9.52)	34.14 (8.94)
	GPs, mean (SD)^b,c,e^	8.32 (2.98)	9.14 (2.48)	8.58 (2.67)	9.50 (2.05)	8.37 (2.84)	8.84 (2.69)	9.48 (2.40)	9.99 (2.05)
	Counselors, mean (SD)	7.88 (2.79)	8.75 (2.61)	8.25 (2.73)	8.94 (2.61)	8.46 (2.97)	8.60 (2.57)	9.06 (2.82)	9.29 (2.50)
	Clinical psychologists, mean (SD)^b,c^	5.72 (3.46)	6.66 (3.30)	5.92 (3.47)	6.99 (3.29)	6.42 (3.53)	6.73 (3.73)	6.61 (3.46)	7.79 (3.15)
	Psychiatrists, mean (SD)^b^	5.60 (3.33)	6.53 (3.36)	5.69 (3.15)	6.85 (3.01)	5.96 (3.25)	6.18 (3.54)	6.39 (3.14)	7.08 (2.88)
**Symptoms of depression**									
	CES-D score, mean (SD)^c^	10.2 (6.22)	9.4 (7.15)	10.09 (8.26)	7.95 (6.79)	21.83 (9.27)	21.56 (10.73)	22.51 (9.87)	22.26 (10.84)

^a^ All values are number (%) unless otherwise indicated.

^b^ Significant effect of wave.

^c^ Significant effect of distress group.

^d^ Significant interaction of condition and wave.

^e^ Significant effect of condition.

## Discussion

This study used a randomized controlled trial to evaluate health e-cards as a means of encouraging help seeking for depression among young adults. We found no significant differences between the two depression information interventions. Neither of these interventions was more effective than a control condition in increasing formal help-seeking behavior or intentions, or in improving beliefs about depression treatments, recognition of depression, knowledge of the help-seeking process, or depressive symptoms, but they were associated with more positive beliefs about formal help sources. At post-intervention, participants in the two active arms and the control condition reported higher intentions of seeking help for depression from a GP or mental health professional, were more likely to rate interpersonal therapy as being helpful, and also reported being more knowledgeable about the help-seeking process. Depression information had no significant effect on informal help-seeking behavior, intentions, or beliefs.

### Strengths and Limitations

This study is one of few to have used a randomized controlled trial to evaluate the effectiveness of depression information interventions for increasing help-seeking behavior [5-7] and the first to use an e-card modality for delivering the intervention. The study had several strengths in its design: the inclusion of a group of individuals experiencing high levels of distress who could be considered in need of help, comparison of the depression information intervention to a control condition that did not provide information about depression, presentation of the trial as a health and well-being study rather than a depression study, and the use of Web logs to objectively track individuals’ adherence to delivered materials rather than relying on self-report methods.

The principal limitation of the study is that recruitment rates were low. A further limitation is that the recruitment methodology is likely to have selected for participants with a heightened interest in the research topic and willingness to participate (a problem that is pandemic to most public health trials attempting to ascertain participants from a representative community catchment). As a consequence, results may not be generalizable to the whole population of young adults.

Another possible limitation of the study is that the follow-up period of 3 weeks post-intervention may not have been a sufficient amount of time to capture subsequent changes in actual help-seeking behavior. Even if an individual is willing to seek help for depression, other factors such as availability of appropriate help sources can delay help seeking [12]. Ideally, the study would have included a second follow-up questionnaire that assessed outcome changes over a longer period of time (eg, 3 months).

A high proportion of individuals in the high distress group reported at baseline that they were currently receiving care for depression from a GP or mental health professional and/or had previously sought help for depression. This is a potential problem since a help-seeking intervention is unlikely to increase help seeking in those already in treatment or in those already with experience of health services. To address this potential weakness, a subsidiary analysis of help-seeking behavior was conducted with these individuals excluded. The findings remained unchanged with this group removed. The results from this analysis should, however, be interpreted as exploratory due to the low participant numbers and increased risk of type 2 error.

### Implications of the Study

The analysis of the effect of condition on help-seeking behavior revealed no significant differences between the basic and enhanced conditions, or between the combined depression information condition and control condition. This may be attributable to the smaller than planned sample, which reduced power. However, the confidence intervals of the nonsignificant difference between the depression information intervention and control arms imply the maximum possible effect is unlikely to exceed 13% for formal sources and 25% for informal sources in the high distress group. Given that professional help of some sort is indicated for this group, there is a need to refine and further develop these brief interventions with a view to increasing their potential effectiveness before conducting a larger trial.

An idea for improving the effectiveness of health e-cards would be to modify their content to describe help seeking for mental health problems in general, rather than just depression. In Australia, anxiety disorders are more prevalent among young adults than affective disorders [1], and given this study’s use of the K10 as a screening instrument rather than a more specific measure of depression, it is reasonable to assume that some participants in the high distress group might have been experiencing anxiety in addition to, or, rather than, depression. The intervention’s focus on depression might have caused these participants to feel that the information and questions about help seeking for depression were not relevant to them, reducing the effectiveness of the e-cards in improving help seeking.

As they stand, the study’s findings are consistent with previous randomized controlled trials that have also failed to demonstrate increased help seeking from health professionals following depression information interventions [5-7]. We did, however, find a significant effect of occasion of measurement (post-test versus pre-test) on formal help-seeking *intentions* in all conditions. At post-intervention, participants in both the depression information and control conditions were more likely to report higher intentions of seeking help for depression from a GP or mental health professional. This finding may suggest that the act of making contact with young adults and involving them in an intervention may have been more influential in modifying help-seeking intentions than the informational content provided in the intervention. Although the contact was automated and personalization was minimal, some participants may have felt a connection with the researcher and, as a result, formed more positive attitudes about seeking help from other health professionals in the future. An alternate explanation is that the completion of survey questions about health and well-being led some participants to become more aware of problems they might have been experiencing and influenced their perceived need for help. Participants in both conditions spent a considerable amount of time attending to questions about general health topics and depression. Parslow et al [34] have previously found that participants’ use of GP services increased in the 3-month period after participating in a community-based epidemiological health survey. A major limitation to either of these interpretations is the lack of a wait list or nonintervention control. Without these, we can not rule out the possibility that a lapse of time alone yielded greater help-seeking intentions.

### Conclusions

There is a need to identify interventions that increase help seeking for depression among young adults. This study failed to find evidence that providing depression information in the form of brief e-cards encourages such help seeking. Further research is required to investigate potential variables that might be critical in facilitating help seeking and in redesigning the content of the e-cards. It may be significant that involvement in the study was associated with increased help-seeking intentions among participants in all conditions. This suggests that mechanisms other than depression information in brief interventions may increase help seeking, but further investigation is required to explore this possibility and identify the nature of such mechanisms.

## Multimedia Appendix

Emails sent to participants in each condition with embedded URLs to health e-card websites

## Abbreviations

AHSQ: Actual Help Seeking Questionnaire
CES-D: Center for Epidemiological Studies Depression Scale
GHSQ: General Help Seeking Questionnaire
GP: general practitioner
K10: Kessler Psychological Distress Scale
