# Supplementary material for: Health e-Cards as a Means of Encouraging Help Seeking for Depression Among Young Adults: Randomized Controlled Trial
Source: J Med Internet Res. 2009 Oct 22;11(4):e42. doi: 10.2196/jmir.1294 (PMC2802564; doi:10.2196/jmir.1294)
Supplement: Supplementary file 1 [file jmir_v11i4e42_app1.pdf]

APPENDIX 1: Emails sent to participants in each condition with embedded URLs to "health e-card" web sites.

To view the health e-card sites, click on the URL link or copy and paste the address into your browser. Since completion of the trial, sites have been archived using WebCite®. As a result, some links within the sites are no longer functional.

Basic Intervention: Health e-card #1

From: [researcher's email address]
To: [participant's email address]
Subject: Health e-card #1

Hi [participant's first name],

This is Daniel from the ANU Health e-card Project.

Over the next three weeks, you'll receive a series of Health e-cards that aims to provide clear and practical information about depression.

Today's health e-card will help you to recognise the signs and symptoms of depression.

To view your Health e-card, click on the link below or copy and paste the address into your browser:

http://www.webcitation.org/query?url=http%3A%2F%2Fwww.ehub.anu.edu.au%2Fecard%2Fintro.php&date=2009-06-16

Best wishes,

Daniel Costin
ANU Health e-card Project

ANU College of Medicine and Health Sciences
Building 63
The Australian National University
Canberra ACT 0200 Australia
E: [researcher's email address]

Basic Intervention: Health e-card #2

From: [researcher’s email address]
To: [participant’s email address]
Subject: Health e-card #2

Hi [participant’s first name],

This is Daniel from the ANU Health e-card Project.

Today's Health e-card will help develop your understanding of depression.

To view your Health e-card, click on the link below or copy and paste the address into your browser:

http://www.webcitation.org/query?url=http%3A%2F%2Fwww.ehub.anu.edu.au%2Fecard%2Ftom.php&date=2009-06-16

Best wishes,

Daniel Costin
ANU Health e-card Project

ANU College of Medicine and Health Sciences
Building 63
The Australian National University
Canberra ACT 0200 Australia
E: [researcher’s email address]

Basic Intervention: Health e-card #3

From: [researcher’s email address]
To: [participant’s email address]
Subject: Health e-card #3

Hi [participant’s first name],

This is Daniel from the ANU Health e-card Project.

Today's Health e-card aims to develop your understanding of the type of help / treatment that different health professionals provide for depression. I'll also provide you with some tools and resources that will come in handy if you or a mate ever need to get help.

To view your Health e-card, click on the link below or copy and paste the address into your browser:

http://www.webcitation.org/query?url=http%3A%2F%2Fwww.ehub.anu.edu.au%2Fecard%2Fhelp_info.php&date=2009-06-16

Best wishes,

Daniel Costin
ANU Health e-card Project

ANU College of Medicine and Health Sciences
Building 63
The Australian National University
Canberra ACT 0200 Australia
E: [researcher’s email address]

Enhanced Intervention: Health e-card #1

From: [researcher’s email address]
To: [participant’s email address]
Subject: Health e-card #1

Hi [participant’s first name],

This is Daniel from the ANU Health e-card Project.

Over the next three weeks, you'll receive a series of Health e-cards that aims to provide clear and practical information about depression.

Today's health e-card will help you to recognise the signs and symptoms of depression.

To view your Health e-card, click on the link below or copy and paste the address into your browser:

http://www.webcitation.org/query?url=http%3A%2F%2Fwww.ehub.anu.edu.au%2Fecard%2Fdepression.php&date=2009-06-16

Best wishes,

Daniel Costin
ANU Health e-card Project

ANU College of Medicine and Health Sciences
Building 63
The Australian National University
Canberra ACT 0200 Australia
E: [researcher’s email address]

Enhanced Intervention: Health e-card #2

From: [researcher's email address]
To: [participant's email address]
Subject: Health e-card #2

Hi [participant's first name],

This is Daniel from the ANU Health e-card Project.

Today's Health e-card talks about getting help for depression and making decisions about your health and wellbeing that feel right for you.

To view your Health e-card, click on the link below or copy and paste the address into your browser:

http://www.webcitation.org/query?url=http%3A%2F%2Fwww.ehub.anu.edu.au%2Fecard%2Fgetting_help.php&date=2009-06-16

Best wishes,

Daniel Costin
ANU Health e-card Project

ANU College of Medicine and Health Sciences
Building 63
The Australian National University
Canberra ACT 0200 Australia
E: [researcher's email address]

Enhanced Intervention: Health e-card #3

From: [researcher's email address]
To: [participant's email address]
Subject: Health e-card #3

Hi [participant's first name],

This is Daniel from the ANU Health e-card Project.

Today's Health e-card aims to develop your understanding of the type of help / treatment that different health professionals provide for depression. I'll also provide you with some tools and resources that will come in handy if you or a mate ever need to get help.

To view your Health e-card, click on the link below or copy and paste the address into your browser:

http://www.webcitation.org/query?url=http%3A%2F%2Fwww.ehub.anu.edu.au%2Fecard%2Ftools.php&date=2009-06-16

Best wishes,

Daniel Costin
ANU Health e-card Project

ANU College of Medicine and Health Sciences
Building 63
The Australian National University
Canberra ACT 0200 Australia
E: [researcher's email address]

Control Condition: Health e-card #1

From: [researcher's email address]
To: [participant's email address]
Subject: Health e-card #1

Hi [participant's first name],

Over the next three weeks, you'll receive a series of Health e-cards that aims to provide clear and practical information about health topics that are relevant for young adults.

You'll receive one Health e-card per week over the next three weeks

Today's Health e-card is about Meningococcal Disease.

To view your Health e-card, click on the link below or copy and paste the address into your browser:

http://www.webcitation.org/query?url=http%3A%2F%2Fwww.ehub.anu.edu.au%2Fecard%2Fmeningococcal.php&date=2009-06-16

Best wishes,

Daniel Costin
ANU Health e-card Project

ANU College of Medicine and Health Sciences
Building 63
The Australian National University
Canberra ACT 0200 Australia
E: [researcher's email address]

Control Condition: Health e-card #2

From: [researcher’s email address]
To: [participant’s email address]
Subject: Health e-card #2

Hi [participant’s first name],

This is Daniel from the ANU Health e-card Project.

Today's Health e-card has some info about Speed and other amphetamines.

To view your Health e-card, click on the link below or copy and paste the address into your browser:

http://www.webcitation.org/query?url=http%3A%2F%2Fwww.ehub.anu.edu.au%2Fecard%2Famphetamines.php&date=2009-06-16

Best wishes,

Daniel Costin
ANU Health e-card Project

ANU College of Medicine and Health Sciences
Building 63
The Australian National University
Canberra ACT 0200 Australia
E: [researcher’s email address]

Control Condition: Health e-card #3

From: [researcher’s email address]
To: [participant’s email address]
Subject: Health e-card #3

Hi [participant’s first name],

This is Daniel from the ANU Health e-card Project.

Today's Health e-card has some info about a drug GHB.

To view your Health e-card, click on the link below or copy and paste the address into your browser:

http://www.webcitation.org/query?url=http%3A%2F%2Fwww.ehub.anu.edu.au%2Fecard%2FGHB.php&date=2009-06-16

Best wishes,

Daniel Costin
ANU Health e-card Project

ANU College of Medicine and Health Sciences
Building 63
The Australian National University
Canberra ACT 0200 Australia
E: [researcher’s email address]
